# Importance of *Propionibacterium acnes* hemolytic activity in human intervertebral discs: A microbiological study

**DOI:** 10.1371/journal.pone.0208144

**Published:** 2018-11-29

**Authors:** Manu N. Capoor, Filip Ruzicka, Gurpreet Sandhu, Jess Rollason, Konstantinos Mavrommatis, Fahad S. Ahmed, Jonathan E. Schmitz, Assaf Raz, Holger Brüggemann, Peter A. Lambert, Vincent A. Fischetti, Ondrej Slaby

**Affiliations:** 1 Laboratory of Bacterial Pathogenesis and Immunology, Rockefeller University, New York, New York, United States of America; 2 Central European Institute of Technology (CEITEC), Masaryk University, Brno, Czech Republic; 3 Department of Microbiology, Faculty of Medicine, Masaryk University, St. Anne's University Hospital, Brno, Czech Republic; 4 Faculty of Health and Life Sciences, School of Life Sciences, Coventry University, Coventry, United Kingdom; 5 Celgene Corporation, Information Knowledge and Utilization, San Francisco, California, United States of America; 6 Department of Pathology, Microbiology and Immunology, Vanderbilt University School of Medicine, Nashville, Tennessee, United States of America; 7 Department of Biomedicine, Aarhus University, Aarhus, Denmark; 8 The School of Life and Health Sciences, Aston University, Aston Triangle, Birmingham, United Kingdom; University of Palermo, ITALY

## Abstract

Most patients with chronic lower back pain (CLBP) exhibit degenerative disc disease. Disc specimens obtained during initial therapeutic discectomies are often infected/colonized with *Propionibacterium acnes*, a Gram-positive commensal of the human skin. Although pain associated with infection is typically ascribed to the body’s inflammatory response, the Gram-positive bacterium *Staphylococcus aureus* was recently observed to directly activate nociceptors by secreting pore-forming α-hemolysins that disrupt neuronal cell membranes. The hemolytic activity of *P*. *acnes* in cultured disc specimens obtained during routine therapeutic discectomies was assessed through incubation on sheep-blood agar. The β-hemolysis pattern displayed by *P*. *acnes* on sheep-blood agar was variable and phylogroup-dependent. Their molecular phylogroups were correlated with their hemolytic patterns. Our findings raise the possibility that pore-forming proteins contribute to the pathogenesis and/or symptomology of chronic *P*. *acnes* disc infections and CLBP, at least in a subset of cases.

## Introduction

Chronic lower back pain (CLBP) is a leading cause of disability among adults worldwide. CLBP has been ranked the number one cause of years lived with disability in the United States [1], and substantial CLBP rates have been reported in Germany (27%) [2], Sweden (33.2%) [3], and Latin America (31.3%) [4]. Most patients with CLBP exhibit degenerative disc disease, an idiopathic condition that predisposes to herniation, sciatica, and significant morbidity/disability. Regarding the underlying mechanism of pain, Freemont *et al*. [5] has demonstrated ingrowth of nerve fibers into degenerated discs (usually accompanied by the presence of annular fissures), representing a potential source of nociception in CLBP, although frank inflammation is often lacking within the paucicellular disc tissue.

In 2001, Stirling *et al*. [6] first published evidence suggesting a relationship between chronic bacterial infection and disc degeneration. A growing body of literature has since argued that disc specimens are often already infected/colonized with *Propionibacterium acnes* at the time of initial therapeutic discectomies (i.e. prior to any previous surgical manipulation). *P*. *acnes* is a prominent Gram-positive bacterial commensal of the human skin and pilosebaceous unit. In multiple independent studies from 2001 to 2017, *P*. *acnes* was isolated from 26% of resected specimens [7–11]. Two 2015 meta-analyses reported a pooled prevalence of bacteria at 34% [12] and 36.2% [13], with *P*. *acnes* as the predominant species. In a recent 368-patient study, Capoor *et al*. [14] confirmed these observations and directly visualized the presence of *P*. *acnes* biofilms *in situ* within resected discs. This result is most consistent with chronic infection.

Chronic infections are often associated with pain symptoms. This type of nociception is classically attributed to body’s inflammatory cascade, a cytokine-induced phenomenon that involves leukocyte infiltration and vascular dilatation. Nevertheless, more direct mechanisms of pathogen-induced pain have also been suggested. For instance, Chiu *et al*. [15] recently demonstrated that *Staphylococcus aureus* can directly activate nociceptor neurons through elaboration of a pore-forming toxin (α-hemolysin) that can generate action potentials in these cells through pathological ion influx. Beyond *S*. *aureus*, bacterial pathogens secrete diverse pore-forming proteins that facilitate cytotoxicity by targeting host-cell membranes [16]. They are often referred to as ‘hemolysins’ for historical reasons—in that erythrocyte lysis (i.e. β-hemolysis) is often observed when these organisms are cultured on blood agar—although these proteins can likewise form pores in other cellular targets (for instance, white blood cells and platelets [17]). In this context, it is noteworthy that hemolytic activity has been associated with cultured strains of *P*. *acnes*, including isolates from orthopedic joint infections [18].

Accordingly, we hypothesized that *P*. *acnes* also expresses pore-forming proteins during chronic intervertebral disc infection/colonization, directly interacting with sensory neurons and contributing to symptoms of CLBP. The current study explores this hypothesis by assessing the hemolytic activity of *P*. *acnes* strains obtained from three independent studies. In the first study, Capoor *et al*. [14] assessed the presence of *P*. *acnes* in degenerated disc tissue obtained from 368 patients who underwent microdiscectomies. Of these, 119 (32.3%) were positive for *P*. *acnes* by culture (>1 colony-forming unit (CFU)/g). Only the 38 *P*. *acnes*-positive disc tissue samples with >1000 CFU/g were examined in this study. In the second study, Rollason *et al*. [19] assessed the presence of *P*. *acnes* in degenerated disc tissue obtained from 64 patients diagnosed with lumbar disc herniation who underwent discectomy surgery. Disc samples obtained from 24 (38%) of these patients tested positive for *P*. *acnes* by culture (1–150 CFU per sample). We were able to obtain 67 separate *P*. *acnes* isolates recovered from these tissues for use in this study. In the third study, Albert *et al*.[20] assessed the presence of *P*. *acnes* in degenerated disc tissue obtained from 61 patients who underwent primary surgery at a single spinal level for disc herniation. Anaerobic cultures were positive in 26 (43%) of these patients. We were able to obtain *P*. *acnes*-positive disc tissue samples for this study.

## Materials and methods

At the time of the three independent studies, Capoor *et al*. [14], Rollason *et al*. [19] and Albert *et al*. [20], each received Ethics Committee approval and the respective studies were approved by the Institutional Review Boards of the participating hospitals. Further, written informed consents were obtained from each patient participating in the respective studies.

### β-Hemolytic activity of disc tissue and disc tissue homogenates

Homogenates (a total of 38) from disc tissue samples obtained during a previous study by Capoor *et al*.[14], along with several solid fragments of intervertebral disc tissue, were directly inoculated on the surface of Wilkins-Chalgren Anaerobe Agar containing 7% sheep's blood and vitamin K (Hi Media Laboratories) and then incubated at 37°C for 14 days under anaerobic conditions. Positive hemolysis was recorded if complete zones of clearance were observed on these plates.

Frozen intervertebral disc tissue samples excised from lumbar disc herniations by Albert *et al*.[20] were used to inoculate plates made using Columbia blood agar containing 7% (v/v) defibrinated sheep’s blood. These plates were incubated as described above. Positive hemolysis was recorded if complete zones of clearance were observed.

### β-Hemolytic activity and phylotyping of P. acnes strains isolated from disc tissue

Fifty-one colonies were selected visually (depending upon the hemolytic characteristics observed) from the 38 *P*. *acnes*-positive disc tissue homogenates studied by Capoor *et al*. [14]. Hemolytic activity was assessed using Columbia Blood Agar Base (Oxoid) supplemented with 7% sheep erythrocytes that had been washed with phosphate-buffered saline. These plates were incubated as described above. Positive hemolysis was recorded if complete zones of clearance were observed on agar plates. These samples were subjected to multiplex PCR typing to determine their phylogroups [21, 22]. Where a sample had both hemolytic and non-β-hemolytic colonies, more than one colony was selected for multiplex PCR typing.

A hemolytic *P*. *acnes* strain from the Collection of Microorganisms (Department of Microbiology, Faculty of Medicine, Masaryk University) isolated phylotype IB from clinical material was used as a positive control to evaluate hemolytic activity. For phylotyping of our isolates, we utilized Nagy *et al*.’s methodology [21], which we tested for accuracy against the set of clinical *P*. *acnes* isolates from the Collection of Microorganisms.

The sixty-seven *P*. *acnes* isolates obtained from Rollason *et al*. [19] had already been subjected to phylogroup analysis using *recA* sequencing and monoclonal antibody typing. Hemolytic activity was assessed using Columbia blood agar supplemented with 7% (v/v) defibrinated sheep’s blood. These plates were incubated as described above. Positive hemolysis was recorded if complete zones of clearance were observed on agar plates.

### Identification of bacteria

Capoor *et al*. utilized matrix-assisted laser desorption/ionization–time of flight mass spectrometry (MALDI-TOF MS) for identification of P. acnes, Rollason *et al*. utilized 16S rRNA-based PCR, and Albert *et al*. utilized (API) biochemical analysis using the Rapid ID 32A kit and by 16S rDNA PCR.

## Results

### β-hemolytic activity observed in cultured disc tissue homogenate

**Fig 1:**
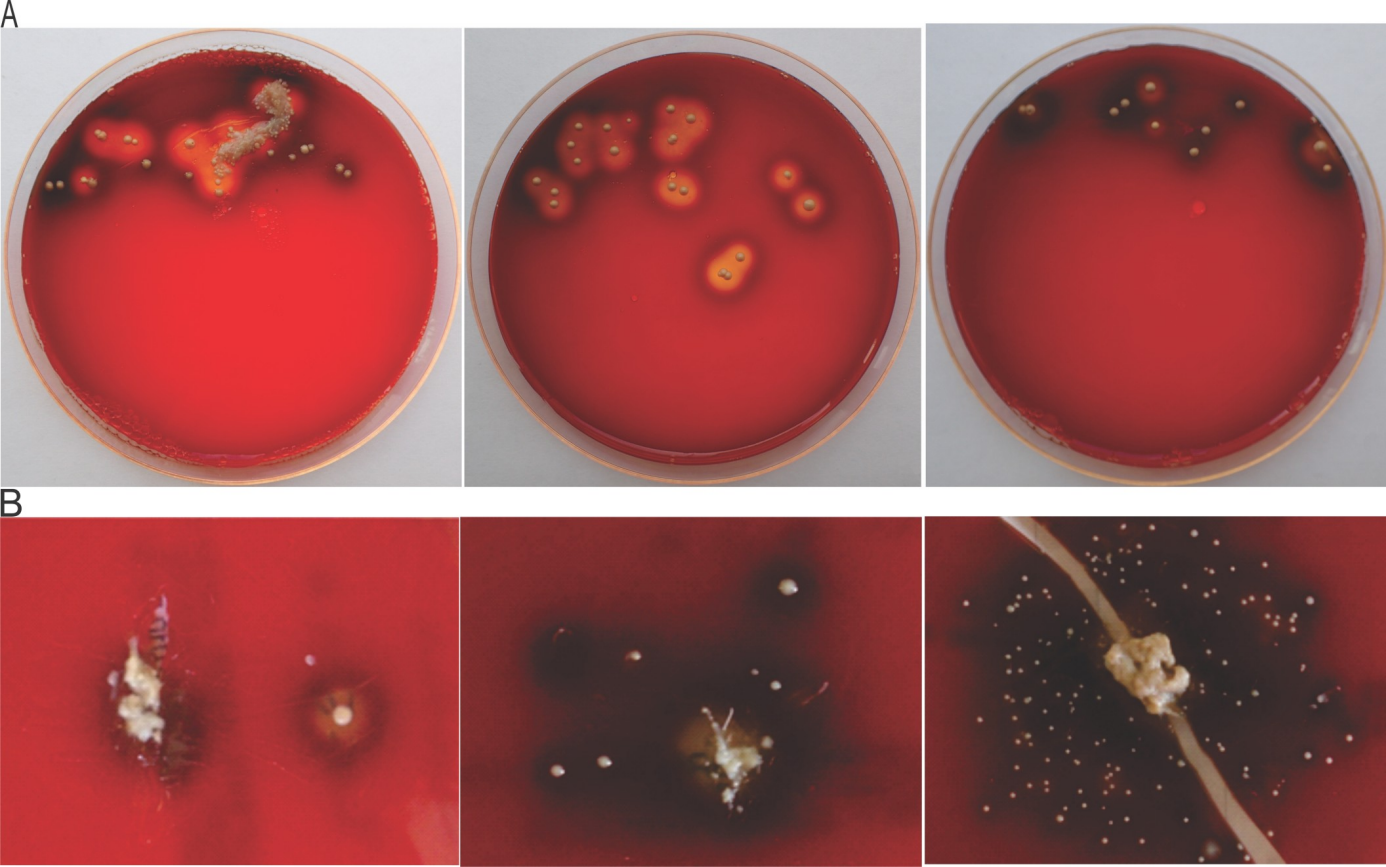
Hemolytic activity of *P*. *acnes* in infected intervertebral disc tissue. Fragments of disc tissue were streaked over the surface of the blood agar plates before pressing and embedding into the agar surface for incubation. Some bacterial colonies (subsequently identified as *P*. *acnes*) show β-hemolysis (clearing of the blood agar), others show no hemolysis and/or production of porphyrins as a dark brown pigmentation with no clearing of the blood agar. (A) Images of hemolysis from intervertebral disc tissue obtained from Capoor *et al*. study [14]. (B) Images of hemolysis from herniated disc tissue received from Albert *et al*. study [20].

Homogenates from the 38 disc tissue samples available from the study by Capoor *et al*. [14] were used to inoculate blood agar plates that were subsequently incubated under anaerobic conditions. Fragments of disc tissue were also streaked over the surface of the blood agar plates before pressing and embedding into the agar surface for incubation. These samples could be most representative of the spinal condition since they were fresh tissue samples excised from patients and cultured the same day. Additional disc tissue samples from the study be Albert *et al*. [20] were similarly used to inoculate blood agar plates. Photographs of representative plates are presented in Fig 1.

After anaerobic culture, 26 (68%) of the 38 plates inoculated with homogenates contained colonies that showed β-hemolysis (clearing of the blood agar) as well as 12 (32%) plates with colonies that displayed no hemolytic activity. Some of the bacterial colonies (subsequently identified as *P*. *acnes*) derived from disc tissue fragments showed β-hemolysis, while others showed either no hemolysis or a brown color likely caused by porphyrins produced by *P*. *acnes*. Of the 26 hemolytic samples, 21 had *P*. *acnes* as the only bacteria isolated from the disc, and 5 samples had additional bacteria cultured (2 *S*. *haemolyticus*, 1 *S*. *pasteuri*, 1 *S*. *warneri*, and one with both *P*. *granulosum* and *F*. *magna*). Of the 12 non-hemolytic samples, 10 had *P*. *acnes* as the only bacteria isolated form the disc, and 2 had additional bacteria cultured (1 *P*. *granulosum* and one 1 *S*. *epidermidus*).

### β-hemolytic activity and phylogroup analysis of P. acnes strains isolated from disc tissue

To assess the *in vitro* hemolytic properties of disc-derived *P*. *acnes* strains, archived isolates from two previous clinical studies were assessed. The results are presented in Table 1.

**Table 1:** Phylogroup-specific distribution of β-hemolysis among *P*. *acnes* isolated from disc tissue.

	*P*. *acnes* strains from Capoor *et al*.[8]		*P*. *acnes* strains from Rollason *et al*.[23]	
Phylogroup	Number of isolates	Number (%) positive for β-hemolysis	Number of isolates	Number (%) positive for β-hemolysis
IA1	20	16 (80%)	19	16 (84%)
IB	10	9 (90%)	6	4 (67%)
IC	1	1 (100%)	0	-
II	20	0 (0%)	35	0 (0%)
III	0	-	7	0 (0%)
Total	51	26 (51%)	67	20 (30%)

A total of 51 morphologically distinct colonies were cultured from the 38 disc specimens obtained from the study of Capoor *et al*. [14] Of note, whenever a single disc specimen yielded both β-hemolytic and non-hemolytic colonies, all colony types from that specimen were selected for inclusion here. Multiplex PCR typing was performed to assign the colonies to the appropriate phylotype. For a majority of these samples, only the visibly dominant strain’s phylogroup was reported. As summarized in Table 1 and broken down according to phylogroup, 26 (51%) of the 51 strains were β-hemolytic (51%). The vast majority of the isolates from phylogroup I (including subgroups IA, IB and IC) were β-hemolytic, but all of the isolates from phylogroup II were non-hemolytic. No isolates from phylogroup III were recovered. The hemolysis pattern from the disc homogenates (Fig 1) corresponded 100% to the hemolysis pattern of the *P*. *acnes* strains subsequently cultured from the same homogenates.

From the independent study of Rollason *et al*. [19], 67 additional disc-derived isolates of *P*. *acnes*, which had bend assigned phylotypes in the original study, were obtained for hemolysis profiling. These isolates were included here irrespective of their original quantitative abundance from disc cultures. As summarized in Table 1, 20 (30%) of the 67 isolates were β-hemolytic. As seen with the samples from Capoor *et al*.’s study [14], the vast majority of the samples from phylogroup I (IA and IB, in this dataset) were β-hemolytic, while all of the strains from phylogroup II were non-hemolytic. Seven strains from phylogroup III were present in this sample set, and all of them were also found to be non-hemolytic.

## Discussion

A substantial portion of the *P*. *acnes* strains tested in this study (46 [39%] of 118) displayed β-hemolytic activity in culture. This is higher than the percentage observed by Wright *et al*. [24], who noted that 22 (21%) of 106 *P*. *acnes* strains obtained from sterile body sites exhibited hemolytic activity with sheep blood. However, it is a lower percentage than that observed by Nodzo *et al*. [18], who found that 13 (59%) of 22 *P*. *acnes* strains isolated from patients who had undergone shoulder arthroplasty exhibited hemolytic activity. Follow-up studies by Boyle *et al*. [25] and Mahylis *et al*. [26] found that 16 (52%) of 31 and 20 (51%) of 39, respectively, isolates from patients who had undergone shoulder arthroplasty exhibited hemolytic activity. When the results of all these studies are added together, 37% of clinical *P*. *acnes* isolates have exhibited hemolytic activity, suggesting that the results of this study are typical of *P*. *acnes* strains isolated from samples obtained in surgical settings.

**Fig 2:**
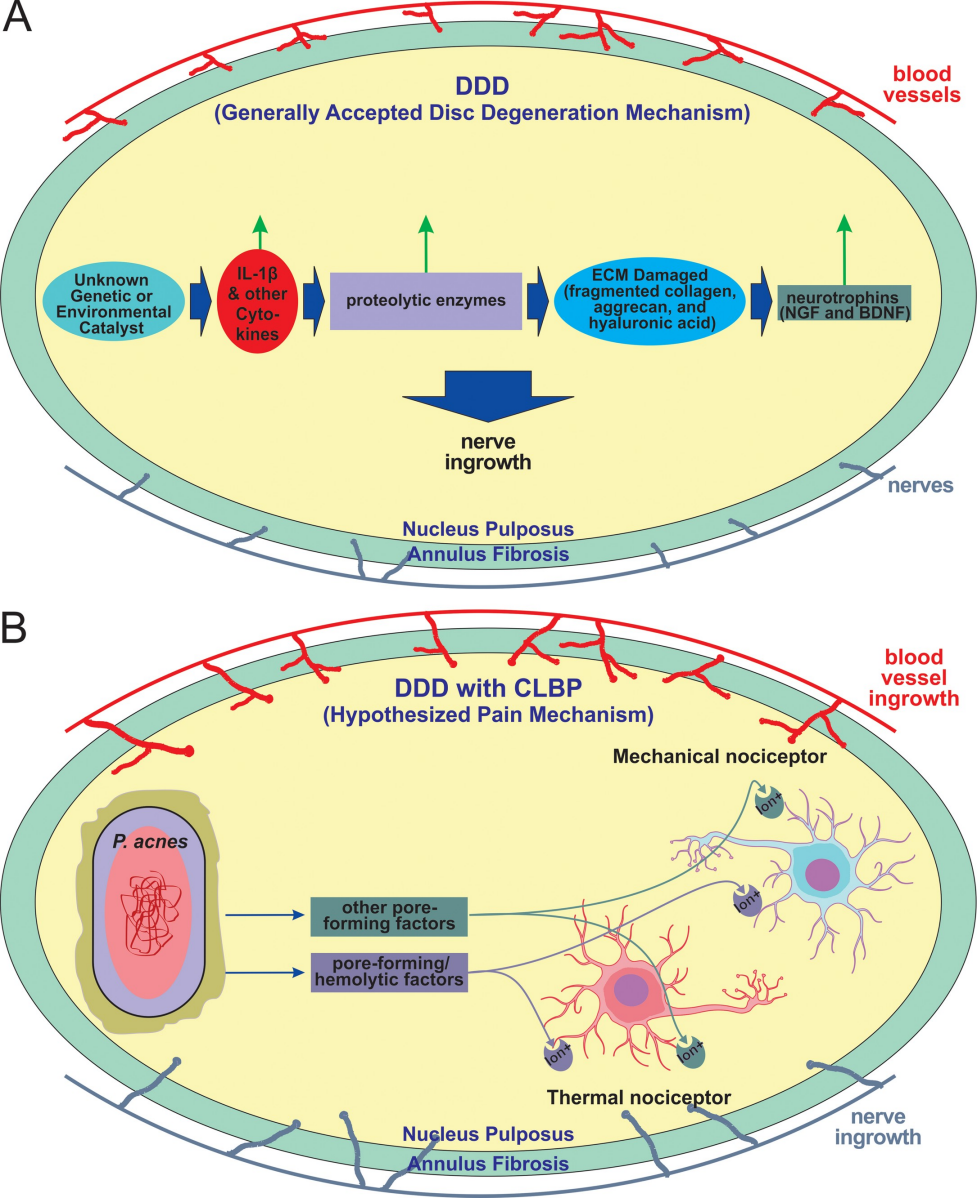
A proposed model underlying CLBP. (A) Generally accepted disc degeneration mechanism. An unknown catalyst is believed to activate the cytokine cascade; IL-1β and other cytokines, in turn, activate proteolytic enzymes that degrade the extracellular matrix (ECM); ECM degradation products (e.g. fragmented collagen, aggrecan, and hyaluronic acid) further activate IL-1β; Positive feedback loops arise; IL-1β stimulates the production of neurotrophins NGF and BDNF, which induce nerve ingrowth. (B) Hypothesized pain mechanism. *P*. *acnes* directly activates nociceptors through pore-forming hemolytic factors and other pore-forming factors that allow the entry of ions that lead to depolarization that induces mechanical and thermal hyperalgesia.

In healthy adult humans and animals, nerves do not extend beyond the outer third of the annulus fibrosus. However, Freemont *et al*. [5] have demonstrated the presence of nerves, including those that express the Substance P (a nociceptive neurotransmitter), in the inner third of the annulus fibrosus, the nucleus pulposus, or both, of patients with chronic low back pain. Infection of these tissues with *P*. *acnes* would place the infecting bacteria in proximity of these ingrown nociceptive nerves, raising the potential for an interaction between P. *acnes* and nociceptive neurons that results in pain (Fig 2). Chiu *et al*. [15] have relatively recently shown, using a mouse model, that *S*. *aureus* induces calcium flux and action potentials in sensory neurons that modulate inflammation through the secretion of N-formylated peptides and the pore-forming toxin α-hemolysin. Mechanical and thermal hyperalgesia in these mice correlated with live bacterial load rather than with tissue swelling or immune activation. Thus, a reasonable mechanism by which chronic *P*. *acnes* infection of one or more intervertebral discs might induce CLBP is the ability of *P*. *acnes* to secrete pore-forming toxins like the α-hemolysin of *S*. *aureus* (Fig 2B). The microbiological findings presented here confirmed that (pore-forming) hemolytic factors are associated with disc tissue, disc homogenates, and *P*. *acnes* isolates obtained from patients undergoing discectomy. These findings support the hypothesis that chronic low back pain may, in some cases, be associated with *P*. *acnes* infection of the intervertebral disc and that this pain may correlated with live bacterial load.

The concept of direct microbe-mediated nociceptor stimulation rather than biomechanical problems being the mode of CLBP-generation would bridge several logical gaps. For one, it would explain how an “undercover” infectious colonization of the very particular physiological and biochemical environment within degenerative human discs could lead to low back pain presenting a clinically obvious pyogenic infection, as would be seen with other pathogens. In addition, the discovery of a dedicated nervous supply to the vertebral body and to the endplates not only provides explanations for how vertebroplasty can reduce fracture-related back pain–it also offers an explanation for how inflammatory endplate-related changes of adjacent bone marrow (Modic Type 1 changes) might correlate with low back pain. A dense nociceptive innervation of the vertebral endplate and the histochemical finding of Substance P in the related afferences [27–29] clearly indicate that not only secondary ingrowth of nerve fibers into degenerative annulus defects but also the vertebral endplate itself are potential targets for such a pain-generating mechanism. An early interventional study [30] demonstrated that so-called discogenic pain can be treated by interruption of the basivertebral nerve. All these data support the need for more research and a better understanding of what chronic low back pain really is and which pathophysiological processes are the most frequent causes.

Phylotyping of the *P*. *acnes* isolates obtained from resected disc tissues showed that isolates from phylogroups II and IA were dominant, while isolates from phylogroups IB, IC and III were present in much smaller numbers. Although these results are somewhat different from the initial observations of McDowell *et al*. [22], who observed a much larger proportion of phylotype III isolates, the results presented here were obtained using a much larger number of disc tissue samples. Hemolytic activity was restricted to those isolates from phylogroup I, including phylogroups IA, IB, and IC; the substantial number of phylogroup II isolates and the small number of phylogroup III isolates showed no hemolytic activity (Table 1). These results are consistent with those of McDowell *et al*. [31] and underline the need for more detailed study of the potential pore-forming toxins of *P*. *acnes* and their phylogroup distributions.

On a true etiologic level, the presence of a *P*. *acnes* toxin (even within a disc) is not complete evidence that it is performing the physiologic function we hypothesize. *In vitro* experiments, in which *P*. *acnes* culture supernatant (or recombinant pore-forming exotoxin) could be applied to a neuronal tissue culture model, are needed to examine the effect of *P*. *acnes* proteins on depolarization. Identifying the genes responsible for the ability of *P*. *acnes* to form pores in neuronal membranes would require knock-in/knock-out experiments with putative hemolysin genes. Mutagenesis models of *P*. *acnes* CAMP genes have been developed [32], and a similar model could be applied to generate *P*. *acnes* hemolysin knockouts, which could be evaluated using designs analogous to those used in the previous animal model studies [15]. Ultimately, such strains might also be investigated in an animal model of *P*. *acnes* discitis [33]. Such a model could be established to assesses “pain” and not simply proliferation of bacteria within the disc.

To investigate when and how hemolysin genes are secreted, the elaboration of pore-forming exotoxins should be studied under diverse growth conditions (e.g. planktonic exponential, planktonic stationary, surface-associated biofilm). Protein expression within a disc may be different from all growth conditions tested, and resected disc tissue would need to be evaluated to represent *in situ* conditions. In addition, clinical studies could be proposed in patients undergoing microdiscectomy for disc herniation. Validated microbiological detection of *P*. *acnes* in the extracted disc tissue would be pursued with the two principal aims being: (i) evaluation of the association between *P*. *acnes* hemolytic activity in the infected disc tissue and pre-operative pain scores, and (ii) determination of the pore-forming factors expressed in the disc tissue specimens and evaluation of their association with pre-operative pain scores.

The presence of genetic code for putative hemolysins in virtually all known *P*. *acnes* genomes, as well as the hitherto not well-appreciated formation of hemolytic zones on microbiological culture plates growing *P*. *acnes* from human discs, should make spinal researchers think deeper and with renewed curiosity. It will be of great importance to test disc material that is obtained during surgery for hemolytic activity in a larger, prospective series. With regard to the suggested use of fresh tissue, a possible limitation of the current study is that it utilized frozen intervertebral disc tissue. If the initial observations presented here were to hold true in these series, such observations would support the pathogenicity of (low-grade infected) nucleus material. It would help explain why in many patients, nucleus fragments that do not exert a significant mechanical compression on the affected nerve root on imaging can still cause such intense radiculopathy.

Future studies could employ hemolytic assays directly on disc tissue material, proteomics using mass spectrometry on the crude disc tissue homogenate to assess whether hemolysins are expressed, mass spectrometry on liquid culture supernatants for secretome analysis, and qRT-PCR or RNA-seq for expression analysis. Hemolysin gene secretion, including assessment of the type I secretion system that does not require signal peptide, could also be studied.
